# Not just how much, but how it’s done: movement activity bout distributions and everyday cognition in older adults with elevated dementia risk

**DOI:** 10.1186/s11556-026-00410-4

**Published:** 2026-04-20

**Authors:** Chih-Hsiang Yang, Jonathan George Hakun, Sunwoo Emma Cho, Jongwon Lee, Halle Prine, Donna Coffman, Rahul Ghosal

**Affiliations:** 1 University of South Carolina, Columbia, USA; 2 The Pennsylvania State University, Hershey, USA

**Keywords:** Ambulatory cognitive assessment, Functional regression model, Activity pattern metrics, Time-varying associations, Brain health, Weekday-weekend contexts

## Abstract

**Background:**

Physical activity (PA) is a protective factor of brain health. Existing literature has predominately examined total PA volume or sedentary time in relation to cognitive health, with limited attention to the distributional features of the movement activity bouts. Variability in the duration of daily movement activity bouts may influence cognitive function beyond total exposure to PA or sedentary time. This study investigates whether daily PA and sedentary bout distributions are associated with daily cognitive function in older adults, and further explores whether these associations differ across the days of the week.

**Methods:**

Older adults (*n* = 87, age = 68.5 ± 7.0 years, 38% male, 21% non-White) with elevated dementia risk completed 14 days of ecological momentary assessment (EMA). Participants wore an accelerometer (activPAL) and completed smartphone-based cognitive assessments of processing speed four times each day. The number and duration of sedentary, light (LPA), or moderate-to-vigorous physical activity (MVPA) bouts (≥ 1 min) were calculated from the activPAL data. Median response time (RT) and number of correct trials from the symbol search task were aggregated at the day level. Two distributional indicators (alpha and Gini) were each calculated for daily sedentary, LPA, and MVPA bouts to investigate their associations with the symbol search outcomes. Functional regression models examined the associations between the distributional indicators and cognitive outcomes by each day of the week (Sunday-Saturday).

**Results:**

On most weekdays, higher Gini (greater variations) in daily MVPA bout length was associated with faster RT (*p*<.001, R^2^=0.138); higher Gini of daily LPA bout length was also associated with more accurate trials (*p*<.05, R^2^=0.022). Conversely, higher Gini in daily sedentary bout length was associated with slower RT (*p*<.05, R^2^=0.164).

**Conclusions:**

This study provides proof-of-concept that the distribution of daily movement behavior bouts is associated with variations in daily cognition. It also illustrates how integrating accelerometry with repeated ambulatory cognitive measures can capture within-person dynamics. The findings can support future research to examine whether specific daily movement patterns may represent appropriate targets for interventions aimed at promoting cognitive health in older adults.

**Supplementary Information:**

The online version contains supplementary material available at 10.1186/s11556-026-00410-4.

## Introduction

Maintaining cognitive health in older adulthood is a critical public health objective, especially given the rapidly aging population and the prevalence of Alzheimer’s Disease and Related Dementias (ADRD) [1]. Accumulating evidence underscores physical activity (PA) as a nonpharmaceutical modifiable factor contributing to sustained cognitive health [2, 3]. Traditionally, PA research has emphasized summary (i.e., scalar) measures, such as total daily activity duration or total steps per day. With the increasing use of wearables and accelerometers in PA research, investigators have begun to examine a variety of PA metrics that characterize the duration, frequency, and variability of individuals’ everyday activity bouts [4, 5]. Activity bouts refer to sustained periods of movement-based activities performed at specific intensities, such as sedentary, light physical activity (LPA), or moderate-to-vigorous physical activity (MVPA) bouts. These activity bout metrics may provide additional predictive value for various health outcomes.

Previous studies linking activity bout data with health outcomes have predominantly applied summary-based metrics, such as total number of daily short (e.g., 10 min) or longer (e.g., 30 min+) MVPA bouts or mean duration (in minutes) of sedentary bouts per day. For instance, a large cohort study found that older adults who had more prolonged sedentary bouts (i.e., lasting ≥ 60 min each time) had poorer physical function, after controlling for their MVPA levels and other key covariates [6]. Another experimental study demonstrated that engaging in both long and short walking bouts (i.e., one bout of 30 min/day or three bouts of 10 min/day, respectively) resulted in comparable and significant increases in VO2 max. However, long walking bouts were more effective than the non-exercise control group in reducing percent body fat and improving mood outcomes [7]. While the summary-based bout measures provide useful insights, they cannot well characterize the distributional features of the activity bouts, which may better reflect individuals’ day-to-day activity dynamics or the underlying mechanisms influencing everyday health outcomes.

Emerging methodological approaches have begun to explore activity bout distributional features in various nuanced ways, including the inequality of activity bout durations (i.e., Gini index) and the distributional pattern of bout lengths (i.e., alpha index) in relation to health outcomes in older populations [8, 9]. The alpha index captures the relative contribution of short versus long bouts, where a higher alpha indicates that short bouts are more predominant and long bouts are rarer within a timeframe (e.g., during waking hours). The Gini index, on the other hand, quantifies inequality in bout lengths, with a higher value indicating that daily physical activity or sedentary bout lengths are more variable rather than being more evenly distributed (less variability) within a timeframe [10]. Evidence from metabolic health research has demonstrated the relevance of these bout distribution features. One study found that adults who accumulated more daily time in prolonged sedentary bouts had lower insulin sensitivity, while spending more time in MVPA bouts lasting 10 min or longer was associated with higher insulin sensitivity [11]. Similarly, older adults who had higher proportion of long sedentary bouts had increased risks of multimorbidity, whereas those with a greater proportion of long LPA bouts appeared to be protective against disability [12].

As the alpha and Gini indices specifically quantify behavioral inequality and fragmentation in the temporal distribution of movement activity bouts. These distributional characteristics of daily activities may contribute to circadian stability, which is not only relevant to metabolic regulation but also to determinants of cognitive function [13–15]. Recent work has shown that the time-of-the-day movement distribution (Gini index) was associated with cross-sectionally assessed cognitive status [16]. Another longitudinal cohort study showed that individuals with cognitive impairment exhibited greater inequality (higher Gini) in their daily activity time compared to those with normal cognition, despite having similar total activity counts and active minutes [17]. These findings highlight that when and how our daily activity is accumulated within and across days may carry more predictive value for cognitive outcomes than overall activity volume alone. Capturing these temporal distribution of daily activity bout thus provides an avenue for further investigation into the underlying mechanisms and dynamic processes that shape cognition [4, 16]. For example, each individual’s activity bout distributions within and across days may reflect distinct underlying biophysiological rhythms that support cognitive health [18]. This line of inquiry remains largely underexplored in the literature, particularly among free-living, community-dwelling older adults.

Further, the existing work is largely based on cross-sectional designs or single-occasion cognitive assessments, which restrict the ability to make inferences on the interindividual differences (stable between-person differences) and the intraindividual variability (i.e., within-person changes over time) between movement activities and cognition. This distinction provides different implications for intervention development and prevention of neurodegenerative diseases. The present study addresses this gap by applying ecological momentary assessment (EMA) of both PA and cognition to understand whether intraindividual variability in daily movement activity distribution (captured by alpha and Gini indices) is associated with intraindividual variability in daily cognition. Unlike traditional one-time, laboratory-based cognitive assessments, EMA captures fluctuations in cognitive performance under real-world contexts, thereby enhancing ecological validity and generalizability [19]. By repeatedly administering brief, validated cognitive tasks in participants’ natural environments, it enables the examination of dynamic, time-sensitive associations between behavior and cognition that cannot be detected using traditional designs.

Another gap in existing research on activity bout distributions and cognitive outcomes is the assumption that these associations are uniform across all days, disregarding potential differences between weekdays and weekend days [20, 21]. This assumption overlooks the evidence suggesting that individuals’ PA patterns vary systematically throughout the week due to lifestyle routines, social engagements, and environmental influences [22, 23]. For example, older adults often maintain structured weekly PA and SB bouts shaped by work schedule, volunteer shifts, healthcare appointments, religious participation, caregiving responsibilities, and social engagement. Variations between weekday and weekend routines may meaningfully influence the distribution of activity bouts and opportunities for cognitive stimulation. Disruptions or dysregulation in older adults' structured weekly activity bout distribution may reflect early functional decline or diminished social participation, both increase the risk of ADRD [24, 25]. Advanced methodologies, such as functional regression models, enables researchers to model daily behavioral patterns and their cognitive correlates as dynamic functions of time [16, 26]. Addressing this gap by examining associations between activity bout distributions and cognition across the week will inform more precisely tailored behavioral interventions considering the day-of-week contexts and influences.

The purpose of the current study was to address current research gaps and establish proof-of-concept by elucidating the dynamic associations between daily distributional features of sedentary, LPA, and MVPA bouts and cognitive function across days of the week. Daily distributional features from the accelerometry data were extracted using the alpha and the Gini indices to model their associations with performance-based cognitive outcomes using functional regressions. For PA bout distributions (LPA and MVPA), we hypothesized that a higher variability in bout lengths (captured via the Gini) and more longer PA bouts (captured via alpha) will be associated with better cognition on a given day of the week, after adjusting for the total number of daily PA bouts; For SB bout distributions, we hypothesized that a higher variability in bout lengths and more longer PA bouts will be associated with worse cognitive performance on a given day of the week, after adjusting for total number of sedentary bouts. Findings can facilitate the identification of optimal days of the week for tailoring interventions to promote cognitive function in older adults’ daily lives.

## Methods

### Participants

The study included 87 community-dwelling older adults (aged ≥ 60 years) from the Midlands region of South Carolina. They were recruited through multiple channels, including study flyers, word-of-mouth referrals, email listservs, and outreach to local senior living facilities and retirement communities. Interested individuals contacted the research team to complete a phone-based eligibility screening. The inclusion criteria included the ability to walk independently and having an elevated risk of ADRD defined as reporting at least one of the following: a family history of ADRD, subjective cognitive decline or memory complaints within the past year, or having body mass index ≥ 25 at enrollment. The BMI-based inclusion criterion was selected based on epidemiological evidence linking overweight/obesity to elevated risk for cognitive decline and ADRD [27]. All criteria used to classify individuals with elevated ADRD risk were informed by the 2020 Lancet Commission report [28]. Exclusion criteria consisted of clinical diagnoses of neurological disorders or mental health conditions (e.g., ADRD, Parkinson’s disease, epilepsy, major depression), having health conditions that limit participation in study protocols (e.g., stroke, severe arthritis), or current participation in other PA or cognitive training intervention(s) that could interfere with study outcomes. During screening, older adults were asked to self-report whether they had ever received a clinical diagnosis of any neurological diseases or major depressive disorder from a healthcare professional. Those who reported a current clinical diagnosis during the phone screening were excluded from participation. Eligible older adults were screened by the staff and subsequently enrolled on a rolling basis after they provided informed consent.

### Ecological momentary assessment protocol

Participants carried an investigator-provided Android smartphone (v9 Pie operating system) programmed with an ecological momentary assessment application, the “Mobile Monitoring of Cognitive Change” or “M2C2” app. The app delivered four EMA prompts each day at semi-random intervals during waking hours, approximately 2.5 h apart starting from each participant’s wake-up time window (e.g., 5am-6am, 6am-7am, etc.). The semi-random scheduling of the four EMAs was selected to balance participant burden with sensitivity to detect within-person cognitive variability [19], and this EMA scheduling scheme is comparable to prior EMA studies using the same smartphone app [29–31]. At each EMA prompt, participants were notified by a 25-sec ringtone with vibration to complete the EMA task within a 30-min time window. During each EMA prompt, three types of ambulatory cognitive assessments were administered (symbol search, grid memory, go-nogo tasks). To establish proof-of-concept, symbol search outcomes were used as the primary measures given that processing speed is a core cognitive domain that is highly sensitive to aging and early dementia-related changes [32, 33].

Participants were instructed to skip EMA tasks if prompts occurred during activities where responding would be unsafe or impractical (e.g., while driving, cooking, bathing). They also wore a research-grade accelerometer (activPAL4) on their right thigh continuously throughout the 14-day protocol, including during sleep and showering time. They were allowed to switch their legs wearing the monitor (e.g., after taking a shower) [34]. During the orientation session, the research staff provided standardized training on proper placement of the activPAL monitor and on using the smartphone to complete the symbol search task. Participants were given sufficient time to familiarize themselves with the cognitive task and were allowed to practice until they felt confident and demonstrated understanding. Participants could receive up to $160 if they provided at least 70% of the EMA and accelerometry data across the 14 days. Prorated compensation was also provided for those with lower levels of compliance. Additional information on the EMA protocol was reported elsewhere [35]. The Institutional Board approved the study protocol and recruitment materials.

### Measures

#### Performance-based cognitive function

The symbol search task is a speeded two-alternative forced-choice test in which participants identify the ‘tile’ at the bottom of the screen (choice sample) that matches one of the tiles displayed at the top (targets). Participants are instructed to make their selection as quickly and accurately as possible. In this study, each symbol search session includes 18 trials, and older adults are able to complete the task within 60 sec typically. The median response time (RT) of all accurate trials and the total number of accuracy trials within a session are the two primary outcomes in the analysis [36]. The symbol search task via the M2C2 app has been validated and implemented in demographically and age-diverse populations [19, 31], and it is sensitive to distinguishing individuals with and without mild cognitive impairment [37]. Example trials of the EMA symbol search task are provided in supplemental Figure S7. Further details regarding the M2C2 cognitive assessments and administration guidelines are available elsewhere [38].

#### Physical activity and sedentary behavior bouts

Participants’ daily movement behaviors were collected via the research-grade activPAL micro4 accelerometers (PAL Technologies, Glasgow, UK) continuously across the 14 days. The activPAL device is widely recognized for its validity and reliability in assessing posture (sitting/lying, standing) and movement (stepping) among older adults under real-world settings [39, 40]. The duration of sedentary, LPA, and MVPA bouts were calculated based on the accelerometry-derived scores of metabolic equivalents (METs) from the PALbatch software to provide proxies of time spent under different activity intensities [41]. Sedentary behavior was defined as < 1.25 METs while in a seated or reclined posture. LPA was defined as 1.25–2.99 METs, and MVPA as ≥ 3 METs. The minimum bout duration was defined as at least one minute of continuous movement activity, and all activity bout outcomes (sedentary, LPA, and MVPA) within the day were used to calculate the alpha and the Gini distribution indices for data analysis [5, 42]. Prior research has also demonstrated high reliability and validity using this research-grade accelerometer to assess weekly movement activities among community-dwelling older adults [43].

#### Covariates

Participants self-reported their key demographics before the 14-day monitoring period to account for the individual differences. Daily valid accelerometry wear time (in hours) and the total number of sedentary, LPA, and MVPA bouts within each day were also calculated as covariates to adjust for variations of data collection time window and activity bouts from day-to-day.

#### Data processing and quality control

To examine the day-level association between movement bout distribution and cognitive function, each participant’s accelerometry data were temporally aligned with their EMA-based cognitive outcome data using same-day timestamps. For a valid day to be included in the analysis, participants need to provide at least one EMA prompt of cognitive task (completed all symbol search trials) and had ≥ 10 h of valid accelerometer wear time. Using these criteria, only ~ 4% of days were excluded from the final analysis due to insufficient accelerometer wear time (< 10 h) or did not answer any EMA prompts on a given day.

### Statistical analysis

The objective of our analysis is to capture the time-varying association between the activity bout distributions and cognitive outcomes across days of the week (Sunday-Saturday). We quantified the activity bouts on each day of the week using the alpha and the Gini indices. All daily distributions were calculated based on activity bouts during participants’ waketime.

To calculate the day-of-week–specific alpha index for sedentary, light, or MVPA bouts, we applied the following equation. The alpha index was computed using all lengths of the sedentary, LPA, or MVPA bouts on a given day of the week (*d*) (1 for Sunday, 2–6 for Monday to Friday, and 7 for Saturday). We used available data from all valid wear days from each participant to define the day-of-week–specific indices.

$$\begin{aligned}\:\alpha\:\left(d\right)&=1+\frac{1}{M\left(d\right)},\:where\:M\left(d\right)\\&=\frac{1}{n}\sum\:_{i=1}^{n}\mathrm{log}\left(\frac{{x}_{i}}{{x}_{m}}\right),\:\:{x}_{i}\in\:\left(d\:th\:day\right).\end{aligned}$$

Here $\:{x}_{i}$ is observed bout length of type of interest (sedentary, LPA, MVPA) and $\:{x}_{m}$ is the shortest recordable bout length. A lower alpha index α(*d*) for a particular activity type would indicate that the person’s activity is consolidated into longer, more sustained bouts of that activity type on that day of the week.

The Gini index (*G*) is a measure of variability (inequality) and can be useful in measuring patterns of accumulation. This is also calculated on a particular day of the week, separately for sedentary, LPA, or MVPA bouts. In particular, the sample Gini index is calculated as half of the relative mean absolute difference of observations on each day of week (*d*) (1 for Sunday, 2–6 for Monday to Friday, and 7 for Saturday), which coincides with the definition based on the Lorenz curve [42].

$$\:G\left(d\right)=\frac{\sum_{i=1}^{n}{\sum}_{j=1}^{n}|{x}_{i}-{x}_{j}|}{2n\sum_{i=1}^{n}{x}_{i}}\:\:\:\:\:\:{x}_{i},{x}_{j}\in\:\left(d\:th\:day\right).$$

The Gini Index takes values in [0,1], a low Gini index closes to zero indicating low variability and low inequality, and a high Gini index closer to 1 indicating high variability (inequality) among the activity bout length distributions. 

The cognitive outcomes used in our model are the two symbol search outcomes: median response time (RT) of all accurate trials and the mean number of correct trials aggregated at the day level, which can be denoted as $\:{Y}_{i}\left(d\right)$ for each outcome for the i-th subject on each day of the week (*d*). Over the 14 days, participants provided two observations for each day of the week (e.g., data from two Mondays, two Tuesdays, etc.), resulting in balanced weekday coverage. This creates day of week-matched observations 

$\:{(Y}_{i}\left(d\right),{\alpha\:}_{i}\left(d\right),{\mathrm{a}\mathrm{n}\mathrm{d}\:G}_{i}\left(d\right))\:$for linking distributions of movement behaviors and same-day cognitive function. Each cognitive outcome $\:{Y}_{i}\left(d\right)\:$is modelled based on $\:{\alpha\:}_{i}\left(d\right)$ and $\:{G}_{i}\left(d\right)$ for each activity bout type (sedentary, LPA, or MVPA) separately, using a flexible function on functional regression model while adjusting for age, sex ($\:{Sex}_{i}=1$ for male, 0 otherwise), education (three levels), and total sedentary/LPA/MVPA bouts ($\:{N}_{i}\left(d\right)$) within each day of the week [44].

1 $$\begin{aligned}{Y}_{i}\left(d\right)&={\beta}_{0}\left(d\right)+{\alpha}_{i}\left(d\right){\beta}_{1}\left(d\right)+{G}_{i}\left(d\right){\beta}_{2}\left(d\right)\\&+{N}_{i}\left(d\right){\beta}_{3}\left(d\right)+Ag{e}_{i}{\beta}_{4}\left(d\right)\\&+{Sex}_{i}{{\beta}_{5}\left(d\right)+Edu_{i}{\beta}_{6}\left(d\right)+\in}_{i}\left(d\right)\end{aligned}$$

The functional model (1) enables estimation and testing of the dynamic associations ($\:{\beta\:}_{j}\left(d\right)$) between activity bout distributions and cognitive outcomes within the week (Sunday-Saturday) while taking into account the correlation of the observations across the week within the same subject. Study day (days 1–14) and study week (weeks 1–2) were not included as covariates because the analysis focused on specific day-of-week effects and each day of the week was represented twice across the 14-day period as noted above. The *Refund* package and *pffr* function in R were used to implement the above functional regression model [45]. For all the functional regression models, the approximate p-value from a Wald-type test and Bayesian confidence intervals was reported for the dynamic regression coefficients [46].

### Power consideration

A Monte-Carlo power analysis was performed to tailor our multilevel data with days nested within participants, assuming a constant functional effect 𝛽 of the activity bout distribution index of interest and using a mixed effects model with random intercept. The simulated outcomes were generated to preserve the intraclass correlations observed for median RT of accurate trials and number of correct trials from the symbol search task. The estimated power for median RT corresponding to *β* = 0.05, *β* = 0.07, and *β* = 0.10 was calculated to be 0.88, 0.99, and 1.00, respectively. Similarly, the estimated power for number of correct trials for 𝛽=0.05, 𝛽=0.07, 𝛽=0.10, are calculated to be 0.66, 0.91, and 1.00, respectively. The simulation results indicated that the applied functional model was adequately powered to detect even small effect sizes.

## Results

### Descriptive statistics

The final analytic dataset comprised 87 older adults with the mean age of 68.5 ± 7.0 years (range = 60–91 years). Among them, 37.9% were male and 20.7% were non-White. They provided a total of 1,170 valid day-level observations linking accelerometry and EMA cognitive outcome data from the same day (mean = 13.4 days/person). Of the 1,170 observations, only 5.2% of days contained a single EMA prompt of cognitive outcome data. The overall compliance rate was high, with 76 of the 87 older adults (87%) provided valid day-level data across all 14 days. Table 1 summarizes the variables included in the model.

**Table 1:** Descriptive summary of the primary variables

Variable	Total (*N* = 87)	Male (*N* = 33)	Female (*N* = 54)
Age (year)	68.5 (7.0)	67.9 (6.3)	68.9 (7.4)
Education (up to high school degree)	17 (19.5%)	3 (9.1%)	14 (25.9%)
Education (some college/bachelor’s degree)	64 (73.6%)	27 (81.8%)	37 (68.5%)
Education (advanced degree)	6 (6.9%)	3 (9.1%)	3 (5.5%)
Symbol search median RT (millisecond)	2327.9 (591.9)	2344.4 (694.3)	2317.8 (526.2)
Symbol search number of correct trials	17.5 (0.4)	17.6 (0.4)	17.5 (0.5)
Number of sedentary bouts per day	28.4 (8.5)	28.1 (7.1)	28.7 (9.3)
Number of LPA bouts per day	34.7 (10.4)	35.4 (9.1)	34.3 (11.2)
Number of MVPA bouts per day	6.9 (4.3)	8.0 (4.3)	6.3 (4.3)

*RT* Response time in milliseconds for accurate symbol search trials

The mean (SD) is shown for the continuous variables and count (%) is shown for the categorical variables. The reported descriptive statistic summaries were averaged across all study days and participants stratified by sex

**Table 2:** Descriptive statistics for daily activity bouts and cognitive outcomes by each day of the week

Day of the week	% of data	Mean (SD) daily sedentary bouts	Mean (SD) daily LPA bouts	Mean (SD) daily MVPA bouts	Mean (SD) symbol search median RT	Mean (SD) correct symbol search trials
Sunday	13.9	27.46 (9.55)	6.23 (5.22)	6.23 (5.22)	2327.8 (592.1)	17.56 (0.5)
Monday	14.6	28.05 (10.20)	7.55 (6.82)	7.55 (6.82)	2330.9 (657)	17.52 (0.5)
Tuesday	14.6	29.72 (9.95)	7.68 (5.22)	7.68 (5.22)	2312 (614.7)	17.51 (0.5)
Wednesday	14.4	28.68 (9.86)	7.08 (5.82)	7.08 (5.82)	2305 (601.1)	17.52 (0.5)
Thursday	14.3	29.32 (10.56)	6.52 (4.74)	6.52 (4.74)	2331.7 (620)	17.53 (0.5)
Friday	14.0	28.59 (10.77)	7.20 (5.94)	7.20 (5.94)	2320.8 (621.2)	17.52 (0.5)
Saturday	14.1	26.52 (9.75)	6.77 (5.30)	6.77 (5.30)	2354.1 (650.1)	17.56 (0.5)

*RT* Response time in milliseconds for accurate trials, *LPA* Light physical activity, *MVPA* Moderate-to-vigorous physical activity

Number of observations = 1170. The reported descriptives are averaged for each day of the week across all participants. Each person provided data for two repetitions of each day of the week across the 14 days

Results from the application of Model (1) were presented for two cognitive outcomes: (i) median RT for accurate trials and (ii) the number of accurate trials in symbol search. Each outcome was modeled with three activity distribution types (sedentary, LPA, and MVPA) as predictors, resulting in a total of six models. In the plots below, we demonstrated findings from the functional regression models that revealed significant time-varying associations between daily activity distribution indices and cognitive outcomes. Additional results showing non-significant or trivial associations were provided in the supplemental file.

### Median response time (RT) and MVPA bout distribution

**Fig. 1:**
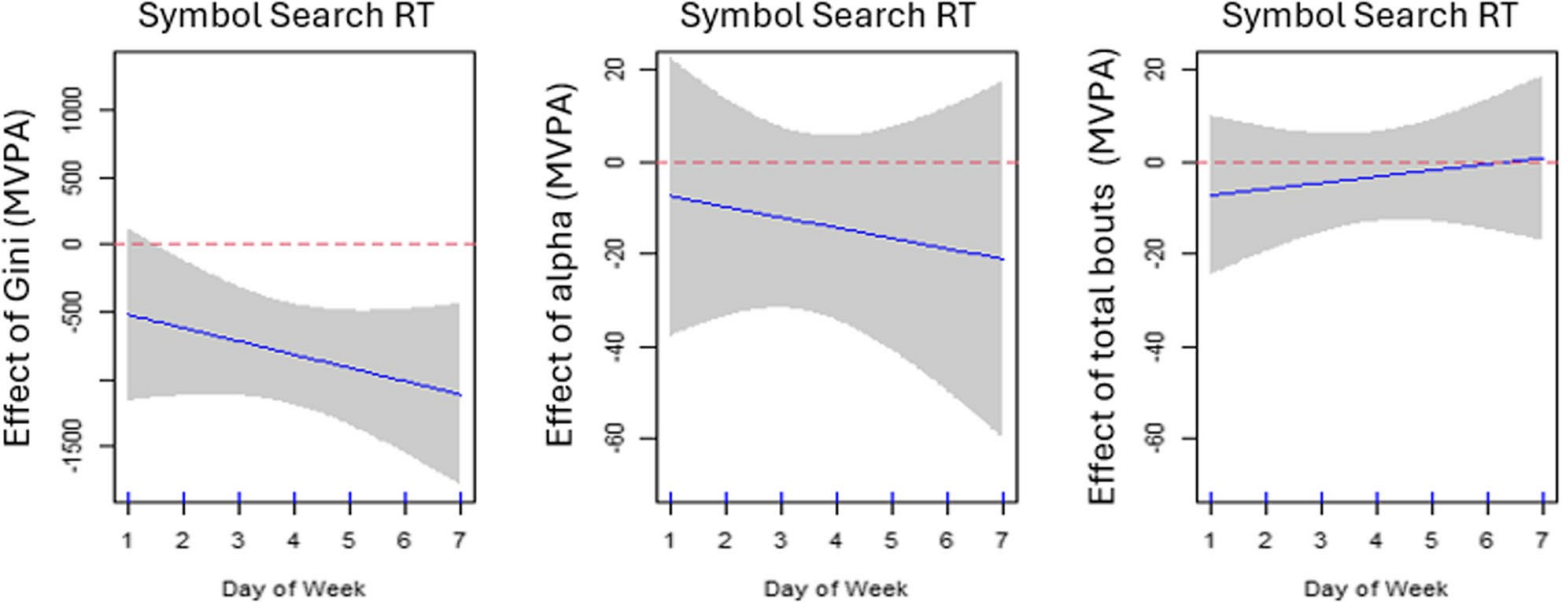
Estimated time-varying associations ($\:{\beta\:}_{j}\left(d\right)$) between median response time (RT) and Gini, alpha, and total number of MVPA bouts. Day of week 1–7 denotes Sunday-Saturday (2–6 are weekdays; 1 and 7 are weekend days). The shaded area represents 95% pointwise confidence intervals of the estimated effects. The negative coefficients in the left plot indicate that a higher Gini is significantly associated with faster RT on most days of the week

Greater variations (a higher Gini index) in daily MVPA bout length on a particular day of week were associated with a faster RT (lower scores) on most weekdays (except Sunday) after adjusting for alpha index, number of MVPA bouts on the same day, age, sex, and education (*p*<.001; adjusted R^2^ = 0.138). The association of RT with alpha index and total number of MVPA bouts was not significant on a particular day after adjusting for other covariates. The estimated day-varying associations are displayed in Fig. 1.

### Number of correct trials and LPA bout distribution

**Fig. 2:**
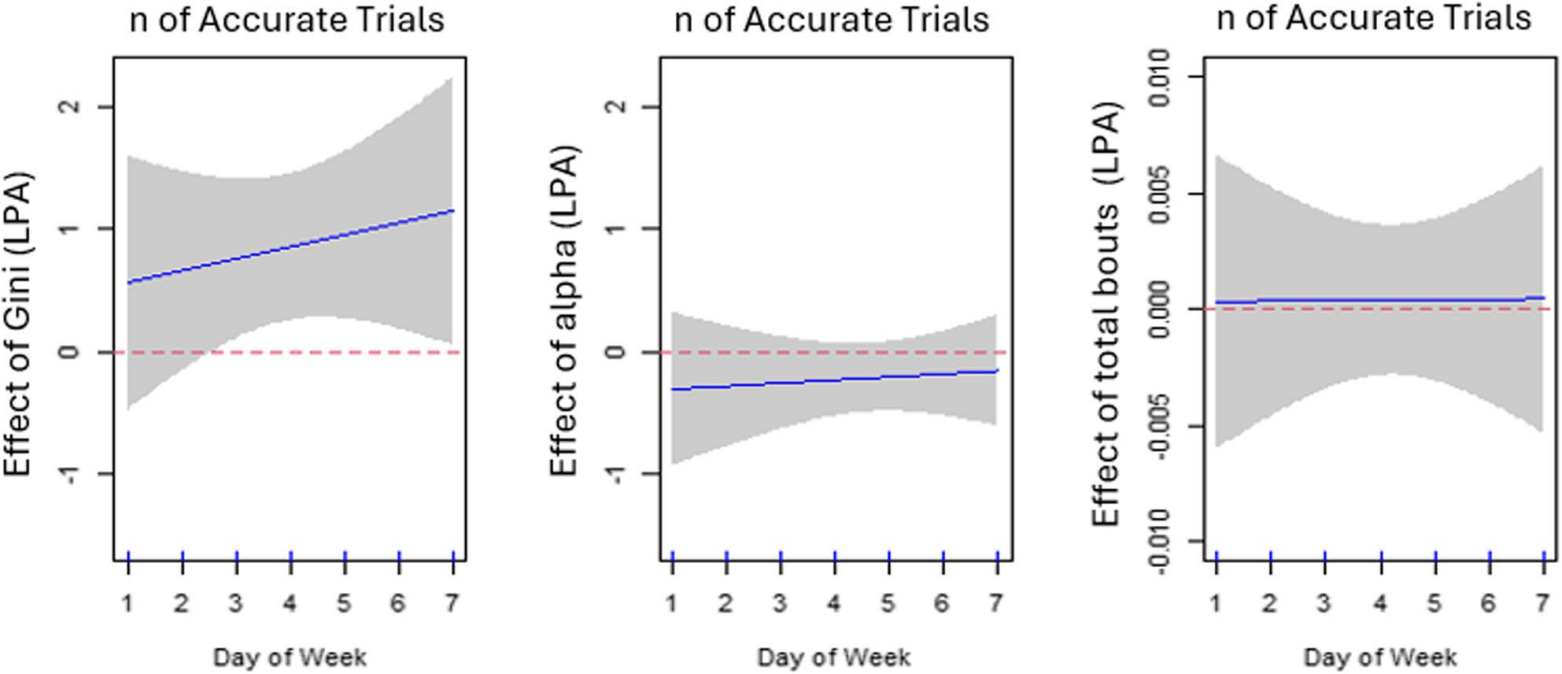
Estimated time-varying associations (${\beta}_{j}\left(d\right)$) between the number of accurate trials and Gini, alpha, and total number of LPA bouts. Day of week 1–7 denotes Sunday-Saturday (2–6 are weekdays; 1 and 7 are weekend days). The shaded area represents 95% pointwise confidence intervals of the estimated effects. The positive coefficients in the left plot indicate that a higher Gini is significantly associated with more accurate trials on most days of the week

Greater variations (a higher Gini index) in daily light physical activity (LPA) bout length were associated with more accurate trials on most weekdays after adjusting for alpha index, total number of LPA bouts on the same day, age, sex, and education (*p*<.05; adjusted R^2^ = 0.022). The association of the number of accurate trials with alpha index and total number of LPA bouts was not significant on a particular day after adjusting for other covariates. The estimated day-varying associations are displayed in Fig. 2.

### Median response time (RT) and sedentary bout distribution

**Fig. 3:**
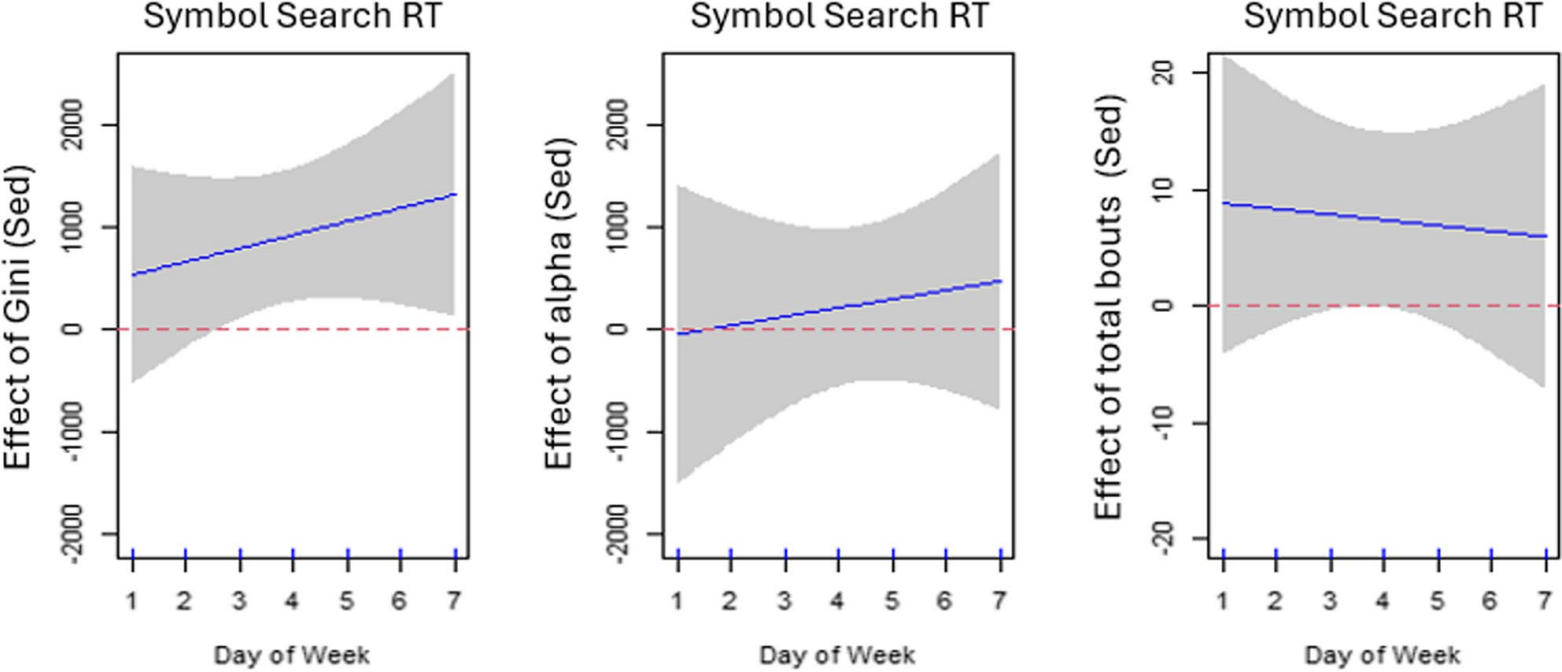
Estimated time-varying associations (${\beta}_{j}\left(d\right)$) between median response time (RT) and Gini, alpha, and total sedentary bouts. Day of week 1–7 denotes Sunday-Saturday (2–6 are weekdays; 1 and 7 are weekend days). The shaded area represents 95% pointwise confidence intervals of the estimated effects. The positive coefficients in the left plot indicate that a higher Gini is significantly associated with longer RT on most days of the week

Greater variations (a higher Gini index) in daily sedentary bout length on a particular day of week were associated with a slower RT (larger value) on most weekdays after adjusting for alpha index, total number of sedentary bouts on the same day, age, sex, and education (*p*<.05; adjusted R^2^ = 0.164). The association of RT with alpha index and total number of sedentary bouts was not significant on a particular day after adjusting for other covariates. The estimated day-varying associations are displayed in Fig. 3.

**Fig. 4:**
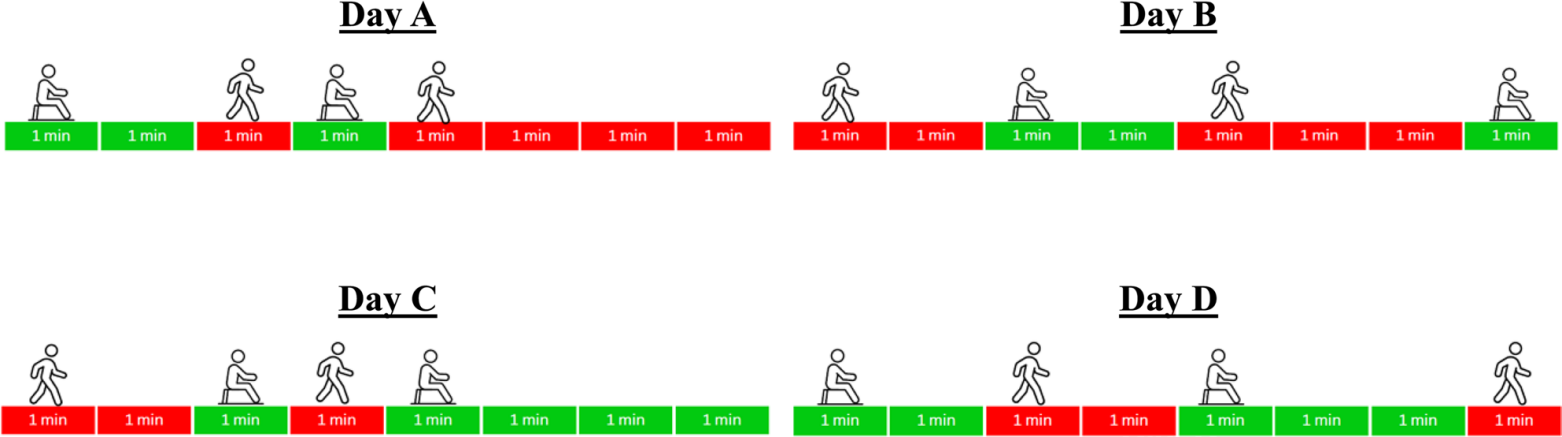
Example scenarios demonstrating differential associations of PA and sedentary bout distributions with daily cognitive function. Figure concept is adapted from Wanigatunga et al. with permission [17]

To illustrate and summarize our findings regarding the Gini index, Fig. 4 presents four simple scenarios to explain which PA and SB bout distributions may be relatively beneficial for faster processing speed. In the upper panel, Day A and Day B have the same average PA bout length (2.5 min) and the same number of PA bouts (*n* = 2). Based on our findings, Day A is associated with better cognitive outcome (i.e., faster RT for MVPA bout distribution or more accurate trials for LPA bout distribution) as it is characterized by greater variability of PA bout length (a higher Gini) compared to Day B. In contrast, in the lower panel, Day C and Day D have the same average SB bout length (2.5 min) and the same number of sedentary bouts (*n* = 2). In this case, Day C is associated with worse cognitive outcome (slower RT) as it has a higher degree of unequal sedentary bout accumulation and the presence of longer uninterrupted sedentary bouts (a higher Gini) compared to Day D. These illustrative scenarios underscore our central findings that greater variability in daily MVPA and LPA bout durations may be associated with more favorable daily cognitive health, whereas greater variability in sedentary bout durations may be associated with poorer cognitive outcomes.

In the supplementary materials, we reported the mean absolute effect sizes of alpha and Gini indices based on different cognitive outcomes (Table S1). Notably, all significant results observed in the current study exhibited relatively large mean absolute effect sizes (≥ 2.1) for the Gini index. The plots for the remaining models with non-significant overall F-tests are shown in Figures S1–S3. Particularly, we did not find any significant association between median RT and LPA bout distributions, and between number of accurate trials and sedentary or MVPA bout distributions. However, the total number of MVPA bouts was positively associated with number of accurate trials across weekdays (see Figure S3). Figure S4 depicted the distributions of alpha and Gini indices for MVPA bouts, stratified by participants with faster versus slower RT on the symbol search task. The alpha distributions were similar across the two groups, whereas the faster RT group showed relatively higher Gini values. Figures S5 and S6 displayed the mean and individual trajectories of daily median RT and number of accurate trials across the study period.

## Discussion

This EMA study offers novel insights into the nuanced associations between daily movement activity bout distributions and performance-based cognitive outcomes among community-dwelling older adults. Results suggest that these associations may vary across different days of the week. During most of the weekdays, greater variations in daily MVPA bout lengths were associated with faster processing speed, whereas greater variations in daily sedentary bout lengths are associated with a slower processing speed. In addition, greater variations in daily LPA bout length were also associated with more accurate trials but not processing speed on most weekdays. These findings underscore the value of using wearable devices to continuously capture nuanced patterns of activity bout distributions linking to cognitive outcomes over time.

To some extent, our findings parallel existing research linking activity bout distributions with various biophysiological outcomes. Prior studies showed that prolonged sedentary bouts were negatively associated with physical function and insulin sensitivity, whereas spending a greater proportion of time in MVPA bouts can mitigate adverse outcomes [11]. Our study further reveals that cognitive outcome may similarly benefit from more evenly distributed sedentary bouts and greater variability in MVPA bouts on most weekdays, as indicated by faster cognitive processing speed. Further, the potential health benefit of long LPA bouts in reducing disability risk is mirrored in the current findings regarding cognitive health [12]. A greater variation of daily LPA bouts (greater Gini) was positively associated with higher cognitive task accuracy. Our findings build on existing work and indicate that variability in activity bout distributions across intensities may signal adaptive patterns linking to healthier profiles, both bio-physiologically and cognitively. In fact, engaging in movement activities are intricately intertwined with multiple systems of the human body (e.g., cardiovascular, respiratory, neural, metabolic, hormonal systems) and therefore could exert widespread influence on diverse health outcomes [47].

One potential mechanism linking PA bout variability to cognitive function is that alternating periods of movement and rest impose repeated physiological transitions that stimulate adaptive regulation across cardiovascular and neural systems [48]. In addition, engagement in varied bout lengths of daily PA may trigger spontaneous brain network communications to support efficient neural processing [49]. Thus, observing higher variability in older adults’ daily activity bouts may reflect underlying physiological and neural adaptability that contributes to cognitive health. On the other hand, older adults with better cognitive health may participate in a wider variety of daily activities with variable bout length, which may also explain the observed positive association between PA bout distribution and cognition [50].

The observed associations between the Gini index and cognitive outcomes were more pronounced on weekdays than on weekends. This may be partly explained by the systematic differences in daily structure and contextual demands across the week. For example, research showed that older adults typically accumulate more PA and sedentary time at outdoor locations on weekdays, whereas weekend days are spent predominantly at home with less contextual variability [51, 52]. In fact, essential out-of-home activities such as healthcare, banking, or visiting public services are generally limited or unavailable on weekend days, which naturally reduces older adults’ opportunities for varied movement and engagement outside their home [52]. As a result, weekend days typically involve more unstructured or passive sedentary behaviors at home, which may lead to reduced variability in movement patterns and fewer cognitively stimulating contexts that weaken the association with cognitive function [53]. In contrast, activities during weekdays often occur in social or goal-directed contexts that foster greater cognitive engagement and regulation, such as running errands, attending appointments, participating in community events. The more structured routines and frequent transitions between active and sedentary bouts on weekdays may therefore reflect the relatively higher weekday bout frequency shown in Table 2. While plausible, these interpretations remain speculative as they were not directly tested. To address this question, future studies should explicitly examine the types of PA and SB that contribute to the weekday vs. weekend day behavioral patterns.

It should be noted that sedentary behaviors may not be uniformly detrimental; rather, its cognitive consequences likely depend on the level of cognitive engagement embedded within the sedentary behavior. Emerging framework has distinguished active sedentary behaviors (e.g., reading, board games, social engagement) from passive sedentary behaviors (e.g., prolonged television viewing, reclining while resting). These subtypes of sedentary behaviors may exert distinct influence on cognitive function and dementia risk through mechanisms such as enhanced (or weakened) neural plasticity, cognitive stimulation, and cerebral blood flow [54]. Although our study cannot differentiate these sedentary subtypes, future research should examine whether cognitively active versus passive sedentary behaviors moderate the associations between movement distribution metrics and cognitive outcomes in older adults.

Nevertheless, this study reveals differing weekday versus weekend associations, highlighting the need to consider temporal dynamics over time rather than assuming invariance of the associations between PA and cognition. Day-of-week contexts may continue to exert meaningful influences on older adults’ daily lives, as participants in this study were mostly retired. Future studies should examine not only the distributions but also the types and locations of PA and sedentary bouts (e.g., through integrating accelerometry with geospatial data) across weekday and weekend contexts. This will clarify how qualitative differences in daily movement patterns are linked to cognition and to inform targeted weekday-weekend interventions for sustaining cognition across the week.

The results provide partial support for our hypotheses - the observed associations between activity bout distribution and cognitive function are exclusively for the Gini index but not the alpha index. These findings may suggest that inequality in the distribution of activity bouts may be more sensitive to cognitive changes than the overall scaling of activity bout durations (i.e., the balance between short and long bouts). In other words, cognitive function in late life may be more sensitive to how variable activity bouts are throughout the day, rather than to whether older adults accumulate longer versus shorter activity bouts. It is also possible that the predominance of very short movement bouts (clustered around 1–2 mins) among older adults in the current study may have restricted the range of alpha values, resulting in limited variability to detect significant associations with cognition. Our findings underscore the potential value of designing interventions to promote a broader mix of PA accumulation, including longer, continuous PA bouts, and breaking up sedentary time into shorter, more evenly distributed bouts may be beneficial for supporting daily cognitive health. For example, using prompts and reminders to break prolonged sedentary periods or engaging in structured LPA and MVPA bouts with all kinds of lengths may offer greater cognitive benefits for older adults.

Our findings further suggest that different cognitive outcomes (i.e., RT vs. accuracy) may also exhibit differential sensitivity to the same activity distribution. Supporting this possibility, a prior study reported that daily MVPA was associated with better processing speed but not with learning or memory among middle-aged adults with cognitive impairment and multiple sclerosis [55]. Recent findings from the landmark Advanced Cognitive Training for Independent and Vital Elderly (ACTIVE) study also found that processing speed training was the only cognitive training modality (compared to the memory and reasoning training arms) linking to the delayed onset of dementia over 20 years in a large US cohort of older adults participating in a structured cognitive training regimen [56]. Different cognitive outcomes may reflect distinct neural pathways in response to the same exposure of movement patterns [57]. Future research should continue investigating which cognitive domains are particularly responsive to the temporal allocation and patterning of daily movement activity bouts. Clarifying these associations represents a valuable direction for future research.

Several limitations of this study warrant consideration. Our 14-day monitoring captured two weeks of daily variability in older adults’ activity bout distributions. Extending the assessment period and increasing weekend sampling in future studies could better characterize weekly movement-pattern consistency to provide a more representative view of older adults’ habitual routines and help inform optimal intervention windows across the week. With longer monitoring periods, the potential learning or fatigue effects on repeated cognitive tasks should be considered. As the current study focuses on the day-of-week effects, we anticipate the learning or fatigue effects to play a less critical role in our findings. In our analysis, each day of the week was represented twice within the 14-day protocol, and participants initiated the study on different days of the week due to rolling recruitment. Thus, it reduces the likelihood that systematic learning or fatigue effects would be confounded with specific weekday patterns. Nevertheless, the possibility of residual practice effects cannot be entirely excluded in our study. Only a limited set of key covariates were accounted in our models. Future research should assess other individual, contextual, and lifestyle factors, such as physical functioning, daily diet, sleep patterns, stress, and environments, to account for their impact on cognition. This study did not assess daytime napping, which is particularly relevant in aging populations. Although the activPAL can detect sitting/lying posture, it cannot accurately distinguish naps from other sedentary behaviors. Consequently, daytime naps may have been classified as sedentary time in our data.

Relevant to this limitation, the current study cannot differentiate sedentary behaviors based on their level of cognitive engagement, thus limiting the interpretation of whether observed associations reflect passive versus cognitively stimulating sedentary bouts. Future studies should incorporate other wearable devices or self-reported measures of daytime naps and different types of sedentary behaviors to evaluate whether these factors alter the observed associations. This study applied diverse criteria to enroll ADRD at risk older adults, including meeting one or more defined risk factors. While this pragmatic definition enhances ecological validity and aligns with existing prevention frameworks aim at targeting broader at-risk populations [58], the heterogeneity of risk profiles may limit direct comparability with studies using more homogeneous or narrowly defined clinical risk groups (e.g., based on APOE ε4 carriers or other biomarker-based criteria). As a related consideration, older adults in this study were mostly well-educated and relatively healthy with adequate mobility. The constrained variability in daily cognitive function may have reduced the sensitivity for the models to detect associations. Studies that include more diverse older adult populations can better account for potential ceiling effects to enhance the generalizability of the findings. Lastly, it is possible that lower daily activity bout variability reflects emerging cognitive changes among older adults at risk for ADRD, rather than serving as a risk factor for future cognitive impairment. Thus, this study cannot rule out the possibility of reverse causality. 

## Conclusions

This EMA study extends aging research by investigating how the intraindividual variability in daily PA and SB distributions are associated with variability in daily cognition. These findings contribute methodologically and conceptually to understanding how the allocation of day-to-day movement activities (not just total volume) may help explain proximal cognitive health in older adults. They also highlight the value of extracting meaningful distributional parameters from high-density mobile and wearable data to advance brain health prevention efforts. Future studies should integrate event-based EMA self-reports or additional sensors (e.g., ambient or sleep trackers) to capture the specific behaviors during sedentary bouts and to disentangle daytime napping from sedentary time, which will enable a more precise characterization of movement behavior–cognition associations in daily life. Continued research in this area will facilitate the development of temporally tailored intervention strategies to sustain everyday cognitive function and reduce neurodegenerative risk in rapidly growing aging populations.

## Supplementary Information

Supplementary Material 1.

## Data Availability

The datasets analyzed in the current study are not publicly accessible to prevent potential re-identification of participants from a geographically limited area, but the presented data and the analytic codes are available from the corresponding author upon reasonable request.

## Abbreviations

ADRD: Alzheimer’s Disease and Related Dementias
PA: Physical activity
LPA: Light Physical activity
MVPA: Moderate-to-vigorous physical activity
EMA: Ecological momentary assessment
M2C2: Mobile Monitoring of Cognitive Change app
RT: Response time
