# Supplementary material for: Not just how much, but how it’s done: movement activity bout distributions and everyday cognition in older adults with elevated dementia risk
Source: Eur Rev Aging Phys Act. 2026 Apr 20;23:19. doi: 10.1186/s11556-026-00410-4 (PMC13227851; doi:10.1186/s11556-026-00410-4)
Supplement: Supplementary file 1 — Supplementary Material 1. [file 11556_2026_410_MOESM1_ESM.docx]

**Supplementary Materials:**

**Table S1: The mean (average) of absolute effect sizes of alpha, Gini indices, and number of bouts, averaged over 7 days of week for all the regression models.**

| Outcome | Movement activity | alpha | Gini | Number of bouts |
| --- | --- | --- | --- | --- |
| Median Response Time (RT) | Sedentary Bout Distribution | 0.43 | **2.16** | 1.52 |
| Median Response Time (RT) | LPA Bout Distribution | 0.37 | 0.11 | 0.63 |
| Median Response Time (RT) | MVPA Bout Distribution | 1.10 | **3.34** | 0.53 |
| Number of Correct Trials | Sedentary Bout Distribution | 1.60 | 0.15 | 1.37 |
| Number of Correct Trials | LPA Bout Distribution | 1.16 | **2.17** | 0.18 |
| Number of Correct Trials | MVPA Bout Distribution | 0.35 | 0.94 | 1.98 |

*medium response time (RT) and LPA bouts:*


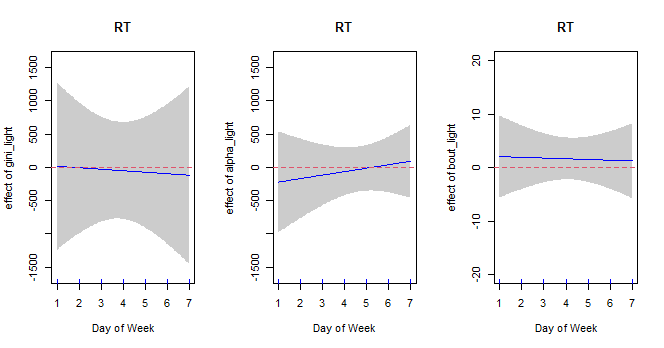


**Figure S1: Estimated time-varying associations (**$\beta_{j}\left( d \right)$**) between medium response time (RT) and Gini, alpha indices of LPA bouts. Day of week 1-7 denotes Sunday-Saturday. The shaded area represents 95% pointwise confidence intervals of the estimated effects.**

*number of correct trials* *and sedentary activity bouts:*


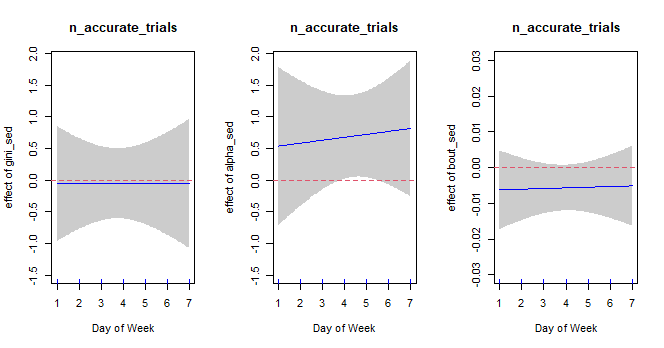


**Figure S2: Estimated time-varying associations (**$\beta_{j}\left( d \right)$**) between number of accurate trials and Gini, alpha indices of sedentary activity bouts. Day of week 1-7 denotes Sunday-Saturday. The shaded area represents 95% pointwise confidence intervals of the estimated effects.**

*number of correct trials* *and MVPA bouts:*


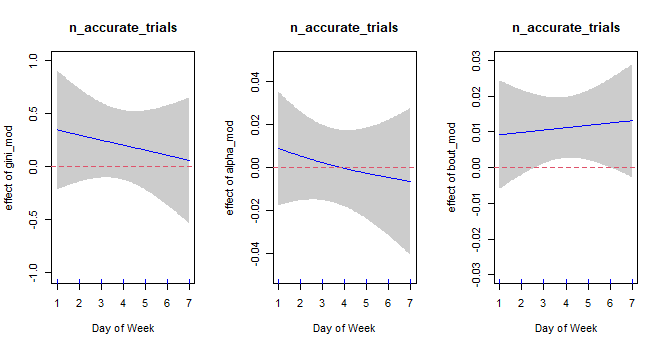


**Figure S3: Estimated time-varying associations (**$\beta_{j}\left( d \right)$**) between number of accurate trials and Gini, alpha indices of MVPA bouts. Day of week 1-7 denotes Sunday-Saturday. The shaded area represents 95% pointwise confidence intervals of the estimated effects.**


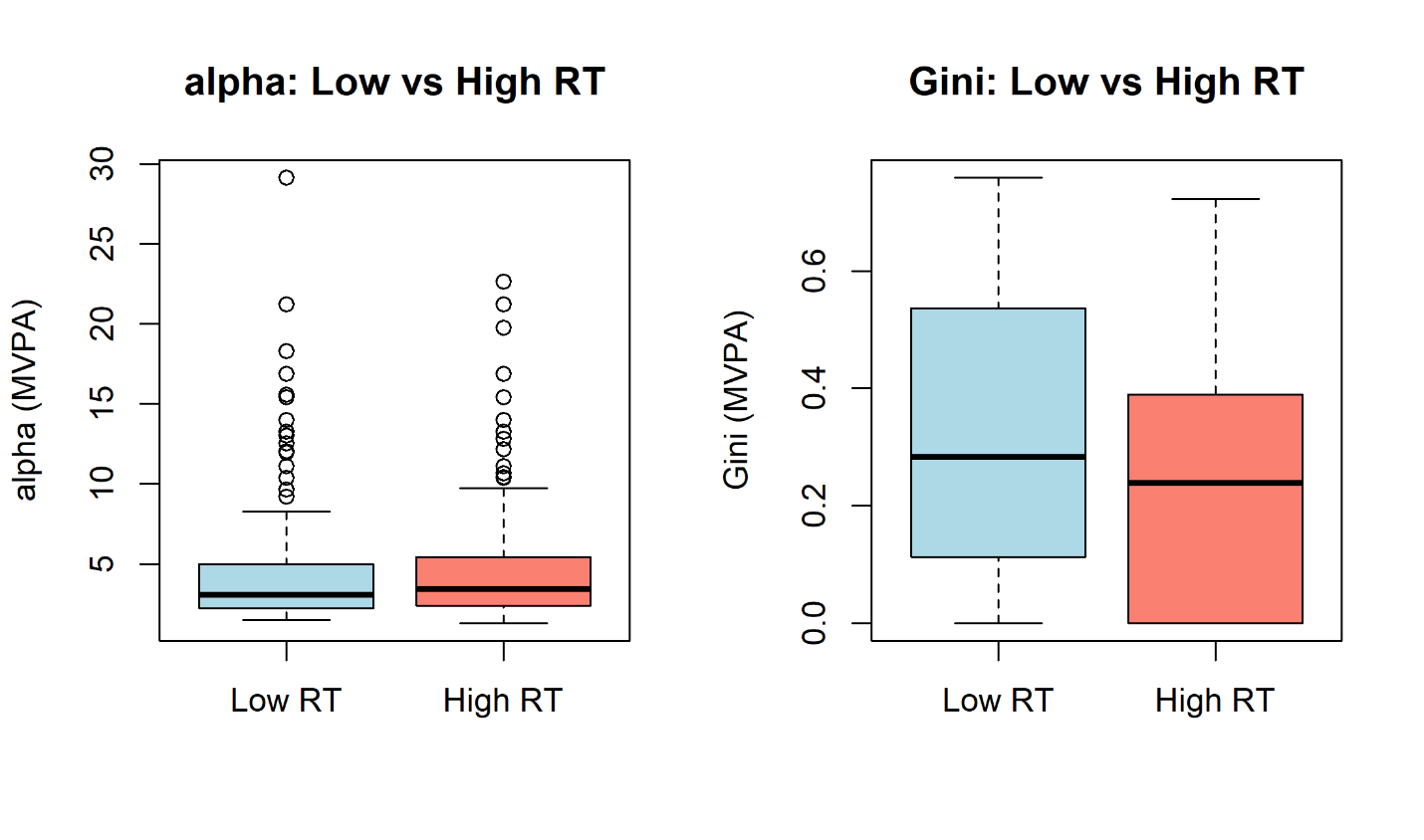


**Figure S4: Distribution of alpha and Gini indices of MVPA bouts for individuals with high RT (> Q3) and low RT (< Q1).**


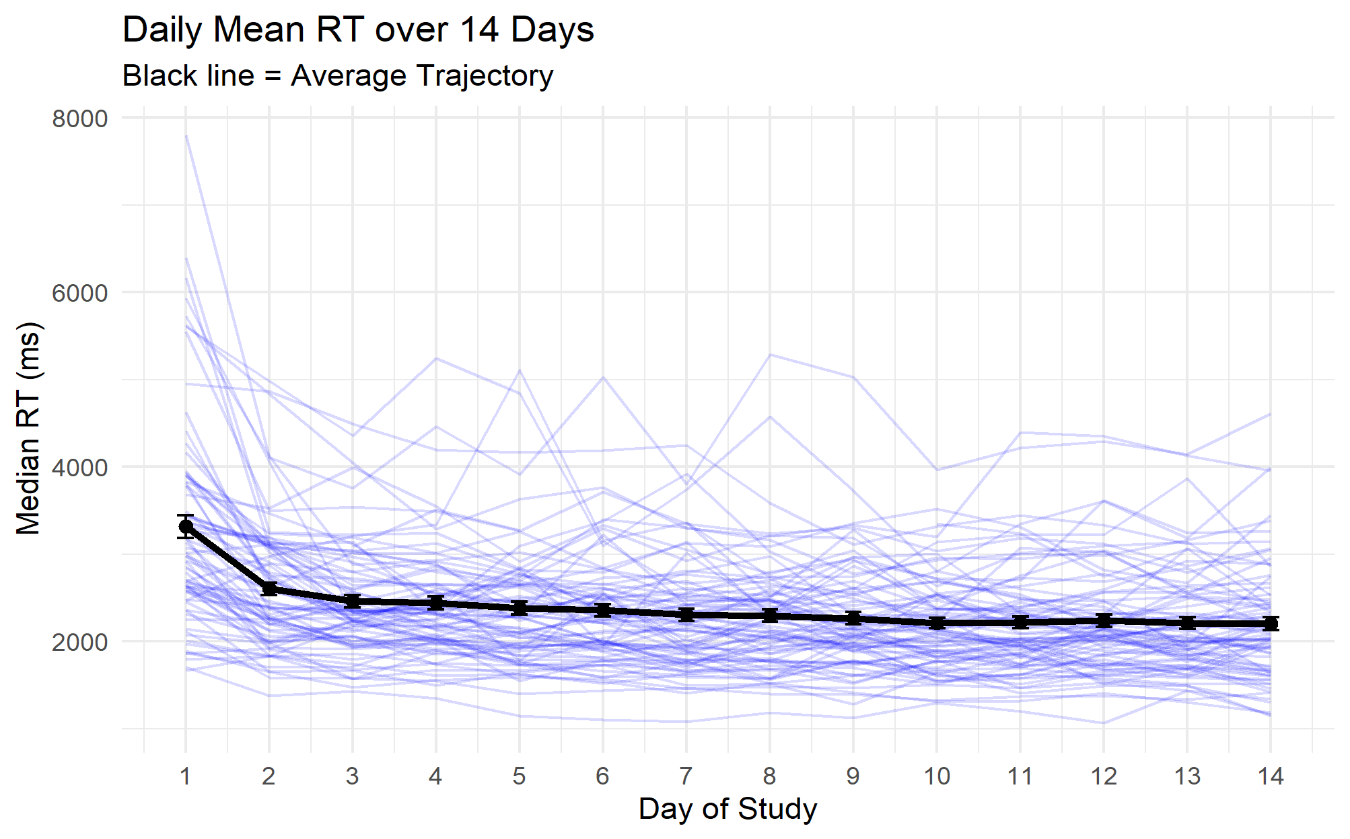


**Figure S5: The individual and average trajectory of median RT across days of study.**


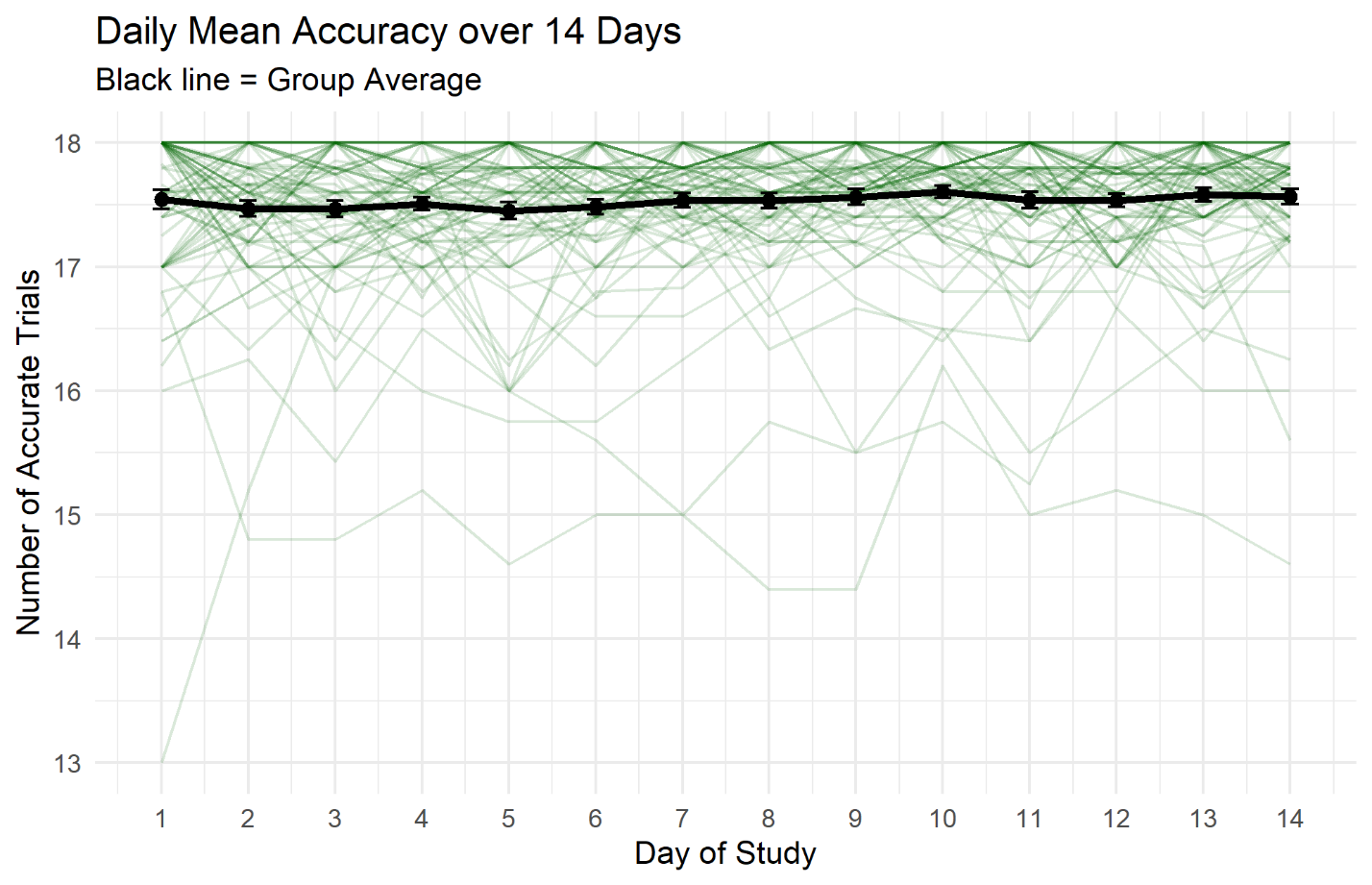


**Figure S6: The individual and average trajectory of number of correct trials across days of study.**

**
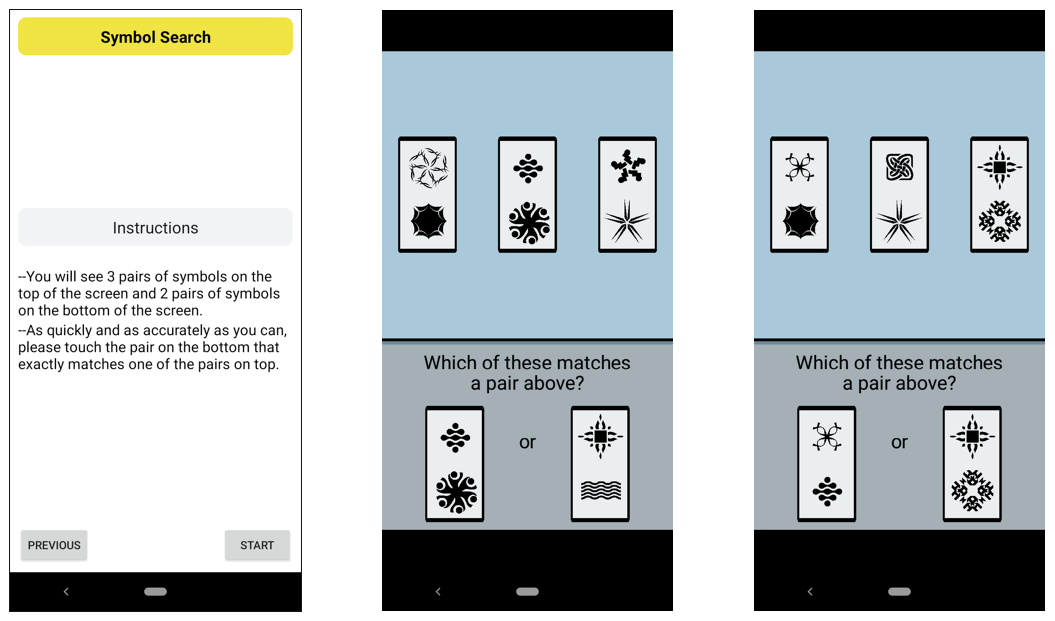
**

**Figure S7: The instructions and example trials of the EMA symbol search task.**
